# Willed action, free will, and the stochastic neurodynamics of decision-making

**DOI:** 10.3389/fnint.2012.00068

**Published:** 2012-09-07

**Authors:** Edmund T. Rolls

**Affiliations:** Oxford Centre for Computational NeuroscienceOxford, UK

**Keywords:** decision, free will, decision-making, the noisy brain, planning, schizophrenia, consciousness, precuneus

## Abstract

It is shown that the randomness of the firing times of neurons in decision-making attractor neuronal networks that is present before the decision cues are applied can cause statistical fluctuations that influence the decision that will be taken. In this rigorous sense, it is possible to partially predict decisions before they are made. This raises issues about free will and determinism. There are many decision-making networks in the brain. Some decision systems operate to choose between gene-specified rewards such as taste, touch, and beauty (in for example the peacock's tail). Other processes capable of planning ahead with multiple steps held in working memory may require correction by higher order thoughts that may involve explicit, conscious, processing. The explicit system can allow the gene-specified rewards not to be selected or deferred. The decisions between the selfish gene-specified rewards, and the explicitly calculated rewards that are in the interests of the individual, the phenotype, may themselves be influenced by noise in the brain. When the explicit planning system does take the decision, it can report on its decision-making, and can provide a causal account rather than a confabulation about the decision process. We might use the terms “willed action” and “free will” to refer to the operation of the planning system that can think ahead over several steps held in working memory with which it can take explicit decisions. Reduced connectivity in some of the default mode cortical regions including the precuneus that are active during self-initiated action appears to be related to the reduction in the sense of self and agency, of causing willed actions, that can be present in schizophrenia.

## Introduction

In this paper, I consider the operation of the different decision-making systems in the brain, and how these are involved in willed action. I also consider the sense of agency which is disturbed in schizophrenia.

## Prediction of decisions from neural activity

Can decisions be predicted from brain activity? When willed actions are self-initiated, it is frequently difficult in neuroimaging studies to determine whether the decision can be predicted from the neural activity, because it is not easy to establish when the decision has been taken. Further, as I consider below, the decision may be taken by brain systems to which we do not have conscious access, and about which we cannot make a verbal report, and this further complicates the answer. In this section, I consider fMRI studies that do aim to predict decisions from brain activity, and then show that in a system that can be studied rigorously, it is possible to predict a decision that will be taken from the noisy (stochastic) fluctuations of neuronal activity in a decision-making network mechanism.

There are fMRI analyses of how early one can predict from neural activity what decision will be taken (Haynes and Rees, 2005a,b, 2006; Pessoa and Padmala, 2005; Lau et al., 2006; Hampton and O'Doherty, 2007; Haynes et al., 2007; Rolls et al., 2009). For example, in one investigation subjects held in mind which of two tasks, addition or subtraction, they intended to perform. It was possible, while they held it in mind in a delay period, to decode or predict with fMRI (functional magnetic resonance neuroimaging) from medial prefrontal cortex activations whether addition or subtraction would later be performed, with accuracies in the order of 70% (where chance was 50%) (Haynes et al., 2007). There is also evidence that the ongoing variations in neural activity measured for example with fMRI may be related to whether a signal is detected and to perceptual decisions (Ress et al., 2000; Boly et al., 2007; Hesselmann et al., 2008, 2010; Sadaghiani et al., 2010).

A problem with such studies is that it is often not possible to know exactly when the decision was taken at the mental level, or when preparation for the decision actually started, so it is difficult to know whether neural activity that precedes an action or report in any way predicts the actual decision that will be taken (Rolls and Deco, 2010). In fMRI studies, the temporal precision is also poor. In these circumstances, is there anything rigorous that our understanding of the neurodynamic mechanisms involved in the decision-making can provide? It turns out that there is, as I show here using an integrate-and-fire attractor network model of decision-making.

**Figure 1:**
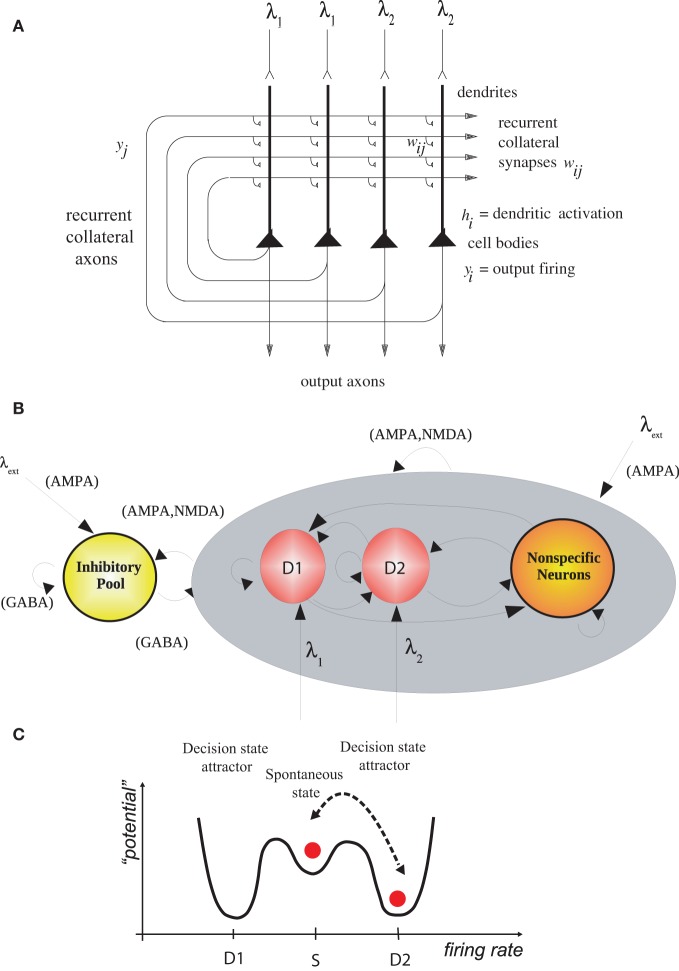
**(A)** Attractor or autoassociation single network architecture for decision-making. The evidence for decision 1 is applied via the λ_1_ inputs, and for decision 2 via the λ_2_ inputs. The synaptic weights *w*_*ij*_ have been associatively modified during training in the presence of λ_1_ and at a different time of λ_2_. When λ_1_ and λ_2_ are applied, each attractor competes through the inhibitory interneurons (not shown), until one wins the competition, and the network falls into one of the high firing rate attractors that represents the decision. The noise in the network caused by the random spiking of the neurons means that on some trials, for given inputs, the neurons in the decision 1 (D1) attractor are more likely to win, and on other trials the neurons in the decision 2 (D2) attractor are more likely to win. This makes the decision-making probabilistic, for, as shown in **(C)**, the noise influences when the system will jump out of the spontaneous firing stable (low energy) state S, and whether it jumps into the high firing state for decision 1 (D1) or decision 2 (D2). **(B)** The architecture of the integrate-and-fire network used to model decision-making (see text). **(C)** A multistable “effective energy landscape” for decision-making with stable states shown as low “potential” basins. Even when the inputs are being applied to the network, the spontaneous firing rate state is stable, and noise provokes transitions into the high firing rate decision attractor state D1 or D2 [see Rolls and Deco (2010)].

We simulated (Rolls and Deco, 2011) an integrate-and-fire attractor network model of decision-making (Wang, 2002; Rolls and Deco, 2010; Deco et al., 2012) with two possible decision states, D1 and D2 (Figure 1). After 2 s of spontaneous firing, decision cues for D1 and D2 were applied to the network. The decision cues for these simulations had equal magnitude, and each decision state was chosen on approximately 50% of the trials, which is the chance performance that was expected. We, however, looked backwards in time to the period before the decision cues were applied, to investigate whether the noisy firing (i.e., variable because each neuron emitted close to Poisson spike trains) before the decision cues were applied in any way was related to which attractor, D1 or D2, won on a particular trial.

**Figure 2:**
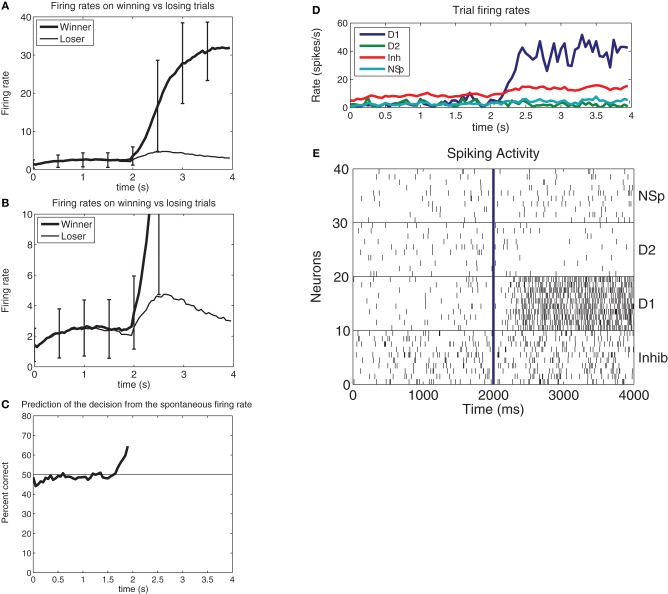
**(A)** Prediction of a decision before the evidence is applied. In this integrate-and-fire simulation of decision-making, the decision cues were turned on at *t* = 2s, with Δ*I* = 0 (i.e., the difference between the decision variables λ_1_ and λ_2_ was 0). The firing rate averaged over approximately 650 winning vs. losing trials for the attractor shows that the firing rate when the attractor will win is on average higher than that for when the attractor will lose at a time that starts in this case at 300 ms before the decision cues are applied. (At *t* = 2s with Δ*I* = 0 the input firing rate on each of the 800 external input synapses onto every neuron of both of the selective attractor populations is increased from 3.00 to 3.04 spikes/s, as described in the text). The error bars show the standard deviation of the firing rate calculated across trials for the 50 ms bins to indicate the noisy operation of this decision-making system. The large standard deviations in the period after the decision cues are applied at 2 s reflect the different decision times of the network on different trials. **(B)** As **(A)**, but with an expanded firing rate axis so that the difference in the firing rates of the pool that will win and of the pool that will lose can be illustrated. **(C)** The prediction accuracy of which pool will win from 100 ms periods of the firing of the two pools at different times before the decision cues are applied at *t* = 2s. The network size was 500 neurons, with 400 excitatory neurons, 400 excitatory recurrent collateral synaptic connections on each neuron, and 40 neurons in each of the two decision pools. **(D)** Example from a single trial of the firing rates of the four populations of neurons for a correct decision (for which Δ*I* = 16). From the top right the plot order is: D1 is the firing rate of the correct and winning attractor D1. Inh is the inhibitory population that uses GABA as a transmitter. NSp is the non-specific population of neurons (see Figure 1). D2 is the firing rate of the correctly losing attractor D2. **(E)**. Rastergrams for the same trial shown in d to illustrate the probabilistic spiking of each neuron. Ten neurons from each of the four pools of neurons are shown. Each vertical line is the spike from a neuron [After Rolls and Deco (2011)].

We showed that in this neurally plausible integrate-and-fire attractor-based model of decision-making (Figure 1), the noise generated by the randomness in the spiking times of neurons can be used to predict a decision for 0.5 s or more before the decision cues are applied (Figure 2). The ongoing noise at the time the decision cues are applied influences which decision will be taken. It is possible to predict on a single trial to more than 68% correct which of two decisions will be taken (Rolls and Deco, 2011). The prediction is made from the spontaneous firing before the decision cues are applied in the two populations of neurons that represent the decisions. Thus, decisions can be partly predicted even before the decision cues are applied, due to noise in the decision-making process.

This analysis has interesting implications for decision-making and free will, for it shows that random neuronal firing times can influence a decision before the evidence for the decision has been provided (Rolls and Deco, 2010, 2011).

## Multiple routes to action

Much perception and action can be performed relatively automatically, without apparent conscious intervention. An example sometimes given is driving a car. Another example is the identification of a visual stimulus that can occur without conscious awareness, in for example, backward masking experiments (Rolls et al., 1994b; Rolls, 2003; Rolls et al., 2005a; Rolls, 2007b, 2011). Another example is much of the sensory processing and actions that involve the dorsal stream of visual processing to the parietal cortex, such as posting a letter through a box at the correct orientation even when one may not be aware of what the object is (Milner and Goodale, 1995; Goodale, 2004; Milner, 2008). Another example is blindsight, in which humans with damage to the visual cortex may be able to point to objects even when they are not aware of seeing an object (Weiskrantz, 1997, 1998). Similar evidence applies to emotions, some of the processing for which can occur without conscious awareness (De Gelder et al., 1999; Phelps and LeDoux, 2005; Rolls, 2005b, 2008a,b; LeDoux, 2008; Brooks et al., 2012; Prabhakaran and Gray, 2012). Further, there is evidence that split-brain patients may not be aware of actions being performed by the “non-dominant” hemisphere (Gazzaniga and LeDoux, 1978; Gazzaniga, 1988, 1995; Cooney and Gazzaniga, 2003). Further evidence consistent with multiple including non-conscious routes to action is that patients with focal brain damage, for example to the prefrontal cortex, may perform actions, yet comment verbally that they should not be performing those actions (Rolls et al., 1994a; Rolls, 1999, 2005b; Hornak et al., 2003, 2004). The actions, which appear to be performed implicitly, with surprise expressed later by the explicit system, include making behavioral responses to a no-longer rewarded visual stimulus in a visual discrimination reversal (Rolls et al., 1994a; Hornak et al., 2004). In both these types of patient, confabulation may occur, in that a verbal account of why the action was performed may be given, and this may not be related at all to the environmental event that actually triggered the action (Gazzaniga and LeDoux, 1978; Gazzaniga, 1988, 1995; Rolls et al., 1994a; Rolls, 2005b; LeDoux, 2008).

This evidence suggests that there are multiple routes to action, only some of which involve conscious processing (Rolls, 2005b, 2008a, 2011, 2013).

The first route is via the brain systems that have been present in non-human primates such as monkeys, and to some extent in other mammals, for millions of years. These systems include the amygdala and, particularly well-developed in primates, the orbitofrontal cortex. These systems control behavior in relation to previous associations of stimuli with reinforcement. The computation which controls the action thus involves assessment of the reinforcement-related value of a stimulus. The representation of the goal and reinforcement outcome provided by the orbitofrontal cortex, and then action-outcome learning in the cingulate cortex, controls behavior in the early stages of learning, and after much training habits involving stimulus-response associations are set up in the basal ganglia (Rolls, 2005b, 2013; Grabenhorst and Rolls, 2011; Rushworth et al., 2011).

**Figure 3:**
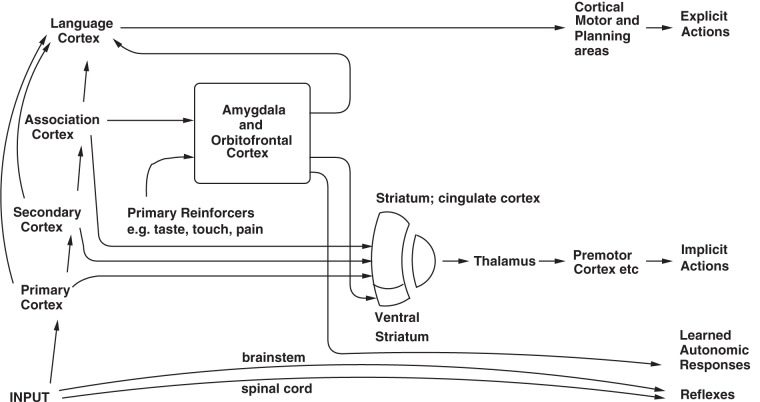
**Dual routes to the initiation of action in response to rewarding and punishing stimuli.** The inputs from different sensory systems to brain structures such as the orbitofrontal cortex and amygdala allow these brain structures to evaluate the reward- or punishment-related value of incoming stimuli, or of remembered stimuli. The different sensory inputs enable evaluations within the orbitofrontal cortex and amygdala based mainly on the primary (unlearned) reinforcement value for taste, touch and olfactory stimuli, and on the secondary (learned) reinforcement value for visual and auditory stimuli. In the case of vision, the “association cortex” which outputs representations of objects to the amygdala and orbitofrontal cortex is the inferior temporal visual cortex. One route for the outputs from these evaluative brain structures is via projections directly to structures such as the basal ganglia (including the striatum and ventral striatum) to enable implicit, direct behavioral responses based on the reward or punishment-related evaluation of the stimuli to be made. The second route is via the language systems of the brain, which allow explicit decisions involving multi-step syntactic planning to be implemented. [After Rolls (2005b)].

The second route in humans involves a computation with many “if … then” statements, to implement a plan to obtain a reward. In this case, the reward may actually be *deferred* as part of the plan, which might involve working first to obtain one reward, and only then to work for a second more highly valued reward, if this was thought to be overall an optimal strategy in terms of resource usage (e.g., time). In this case, syntax is required, because the many symbols (e.g., names of people) that are part of the plan must be correctly linked or bound. Such linking might be of the form: “if A does this, then B is likely to do this, and this will cause C to do this…” The requirement of syntax for this type of planning implies that an output to language systems in the brain is required for this type of planning (see Figure 3). Thus, the explicit language system in humans may allow working for deferred rewards by enabling use of a one-off, individual, plan appropriate for each situation. Another building block for such planning operations in the brain may be the type of short term memory in which the prefrontal cortex is involved. This short term memory may be for example, in non-human primates of where in space a response has just been made. A development of this type of short term response memory system in humans to enable multiple short term memories to be held in place correctly, preferably with the temporal order of the different items in the short term memory coded correctly, may be another building block for the multiple step “if… then” type of computation in order to form a multiple step plan. Such short term memories are implemented in the (dorsolateral and inferior convexity) prefrontal cortex of non-human primates and humans (Goldman-Rakic, 1996; Petrides, 1996; Rolls, 2008b), and may be part of the reason why prefrontal cortex damage impairs planning (Shallice and Burgess, 1996).

Of these two routes (see Figure 3), it is the second that I have suggested above is related to consciousness (Rolls, 2004, 2007a,b, 2011, 2012, 2013). The hypothesis is that consciousness is the state which arises by virtue of having the ability to think about one's own thoughts, which has the adaptive value of enabling one to correct long multi-step syntactic plans. This latter system is thus the one in which explicit, declarative, processing occurs. Processing in this system is frequently associated with reason and rationality, in that many of the consequences of possible actions can be taken into account. I draw a parallel with neural networks: there is a “*credit assignment*” problem in such multi-step syntactic plans, in that if the whole plan fails, how does the system assign credit or blame to particular steps of the plan? [In multilayer neural networks, the credit assignment problem is that if errors are being specified at the output layer, the problem arises about how to propagate back the error to earlier, hidden, layers of the network to assign credit or blame to individual synaptic connections; see Rumelhart et al. (1986); Rolls and Deco (2002) and Rolls (2008b)]. The suggestion is that this is the function of higher order thoughts and is why systems with higher order thoughts evolved. The suggestion I then make is that if a system were doing this type of processing (thinking about its own thoughts), it would then be very plausible that it should feel like something to be doing this. I even suggest to the reader that it is not plausible to suggest that it would not feel like anything to a system if it were doing this.

## Conscious reports of actions initiated by the automatic vs. rational systems

One question that has been discussed is whether there is a causal role for consciousness [e.g., Armstrong and Malcolm (1984)]. The position to which the above arguments lead is that indeed conscious processing does have a causal role in the elicitation of behavior, but only under the set of circumstances when higher order thoughts play a role in correcting or influencing lower order thoughts. The sense in which the consciousness is causal is then it is suggested, that the higher order thought is causally involved in correcting the lower order thought; and that it is a property of the higher order thought system that it feels like something when it is operating. As we have seen, some behavioral responses can be elicited when there is not this type of reflective control of lower order processing, nor indeed any contribution of language (see further, Rolls, 2003; Rolls et al., 2005a; Rolls, 2011, 2012, 2013 for relations between implicit and explicit processing). There are many brain processing routes to output regions, and only one of these involves conscious, verbally represented processing which can later be recalled (see Figure 3).

I suggest that these concepts may help us to understand what is happening in experiments of the type described by Libet and many others (Libet, 2002) in which consciousness appears to follow with a measurable latency the time when a decision was taken. This is what I predict, if the decision is being made by an implicit perhaps reward/emotion or habit-related process, for then the conscious processor confabulates an account of or commentary on the decision, so that inevitably the conscious account follows the decision. On the other hand, I predict that if the rational (multistep, reasoning) route is involved in taking the decision, as it might be during planning, or a multistep task such as mental arithmetic, then the conscious report of when the decision was taken, and behavioral or other objective evidence on when the decision was taken, would correspond much more. Under those circumstances, the brain processing taking the decision would be closely related to consciousness, and it would not be a case of just confabulating or reporting on a decision taken by an implicit processor. It would be of interest to test this hypothesis in a version of Libet's task (Libet, 2002) in which reasoning was required.

Further development of the present proposal, and how it deals with some issues that arise when considering theories of the phenomenal aspects of consciousness, are provided elsewhere (Rolls, 2011).

## Selection between conscious vs. unconscious decision-making mechanisms

The question then arises of how decisions are made in animals such as humans that have both the implicit, direct reward-based, and the explicit, rational, planning systems (see Figure 3) (Rolls, 2008b). One particular situation in which the first, implicit, system may be especially important is when rapid reactions to stimuli with reward or punishment value must be made, for then the direct connections from structures such as the orbitofrontal cortex to the basal ganglia may allow rapid actions (Rolls, 2005b). Another is when there may be too many factors to be taken into account easily by the explicit, rational, planning, system, when the implicit system may be used to guide action. In contrast, when the implicit system continually makes errors, it would then be beneficial for the organism to switch from automatic, direct, action (based on obtaining what the orbitofrontal cortex system decodes as being the most positively reinforcing choice currently available), to the explicit conscious control system which can evaluate with its long-term planning algorithms what action should be performed next. Indeed, it would be adaptive for the explicit system to regularly be assessing performance by the more automatic system, and to switch itself in to control behavior quite frequently, as otherwise the adaptive value of having the explicit system would be less than optimal.

It may be expected that there is often a conflict between these systems, in that the first, implicit, system is able to guide behavior particularly to obtain the greatest immediate reinforcement, guided by genes that specify the goals, i.e., the rewards and punishers. In this case, we can describe our goal behavior as being guided by selfish genes. In contrast, the reasoning or explicit system can potentially enable immediate rewards to be deferred, and longer-term, multi-step, plans to be formed, which may be in the interests of the individual, the phenotype. I have described this as making choices based on selfish genes vs. selfish phenotypes (or selfish phenes).

Now what keeps the decision-making between the “Selfish Genes” and the “Selfish Phenes” more or less under control and in balance? If the second, rational, system chose too often for the interests of the “Selfish Phene,” the genes in that phenotype would not survive over generations. Having these two systems in the same individual will only be stable if their potency is approximately equal, so that sometimes decisions are made with the first route, and sometimes with the second route (Rolls, 2011). If the two types of decision-making, then, compete with approximately equal potency, and sometimes one is chosen, and sometimes the other, then this is exactly the scenario in which stochastic processes in the decision-making mechanism are likely to play an important role in the decision that is taken. The same decision, even with the same evidence, may not be taken each time a decision is made, because of noise in the system.

The system itself may have some properties that help to keep the system operating well. One is that if the second, rational, system tends to dominate the decision-making too much, the first, gene-based emotional system might fight back over generations of selection, and enhance the magnitude of the reward value specified by the genes, so that emotions might actually become stronger as a consequence of them having to compete in the interests of the selfish genes with the rational decision-making process (Rolls, 2011, 2012).

Another property of the system may be that sometimes the rational system cannot gain all the evidence that would be needed to make a rational choice. Under these circumstances the rational system might fail to make a clear decision, and under these circumstances, basing a decision on the gene-specified emotions is an alternative. Indeed, Damasio (1994) argued that under circumstances such as this, emotions might take an important role in decision-making. In this respect, I agree with him, basing my reasons on the arguments above. He called the emotional feelings gut feelings, and, in contrast to me, hypothesized that actual feedback from the gut was involved. His argument seemed to be that if the decision was too complicated for the rational system, then send outputs to the viscera, and whatever is sensed by what they send back could be used in the decision-making, and would account for the conscious feelings of the emotional states. My reading of the evidence is that the feedback from the periphery is not necessary for the emotional decision-making, or for the feelings, nor would it be computationally efficient to put the viscera in the loop given that the information starts from the brain, but that is a matter considered elsewhere (Rolls, 2005b).

Another property of the system is that the interests of the second, rational, system, although involving a different form of computation, should not be too far from those of the gene-defined emotional system, for the arrangement to be stable in evolution by natural selection. One way that this could be facilitated would be if the gene-based goals felt pleasant or unpleasant in the rational system, and in this way contributed to the operation of the second, rational, system. This is something that I propose is the case (Rolls, 2012).

## Mechanisms for decision-making between the implicit and explicit systems

Decision-making as implemented in neural networks in the brain is now becoming understood, and is referred to in section “Prediction of decisions from neural activity”. As shown there, two attractor states, each one corresponding to a decision, compete in an attractor single network with the evidence for each of the decisions acting as biases to each of the attractor states. The non-linear dynamics, and the way in which noise due to the random spiking of neurons makes the decision-making probabilistic, makes this a biologically plausible model of decision-making consistent with much neurophysiological and fMRI data (Wang, 2002; Deco and Rolls, 2006; Deco et al., 2009; Rolls and Deco, 2010). I propose that the same neuronal attractor network mechanism is used in many different decision-making systems in the brain, each present toward the later stages of each hierarchical processing cortical pathway in the cerebral cortex, and each performing categorization of the inputs received (Rolls, 2008b, 2013; Rolls and Deco, 2010; Deco et al., 2012).

I propose (Rolls, 2005b, 2008b) that this model applies to taking decisions between the implicit (unconscious) and explicit (conscious) systems in emotional decision-making, where the two different systems could provide the biasing inputs λ_1_ and λ_2_ to the model. An implication is that noise will influence with probabilistic outcomes which system takes a decision.

When decisions are taken, sometimes confabulation may occur, in that a verbal account of why the action was performed may be given, and this may not be related at all to the environmental event that actually triggered the action (Gazzaniga and LeDoux, 1978; Gazzaniga, 1988, 1995; Rolls, 2005b; LeDoux, 2008). It is accordingly possible that sometimes in normal humans when actions are initiated as a result of processing in a specialized brain region such as those involved in some types of rewarded behavior, the language system may subsequently elaborate a coherent account of why that action was performed (i.e., confabulate). This would be consistent with a general view of brain evolution in which, as areas of the cortex evolve, they are laid on top of existing circuitry connecting inputs to outputs, and in which each level in this hierarchy of separate input-output pathways may control behavior according to the specialized function it can perform.

## Free will and probabilistic decision-making by attractor networks in the brain

These thoughts raise the issue of free will in decision-making.

First, we can note that in so far as the brain operates with some degree of randomness due to the statistical fluctuations produced by the random spiking times of neurons, brain function is to some extent non-deterministic, as defined in terms of these statistical fluctuations. That is, the behavior of the system, and of the individual, can vary from trial to trial based on these statistical fluctuations, in ways that are described in more detail elsewhere (Rolls and Deco, 2010). [Philosophers may wish to argue about different senses of the term deterministic, but is it being used here in a precise, scientific, and quantitative way, which has been clearly defined (Rolls and Deco, 2010)].

Second, do we have free will when both the implicit and the explicit systems have made the choice? Free will would in Rolls' view (2005b, 2008a,b, 2011, 2012) involve the use of language to check many moves ahead on a number of possible series of actions and their outcomes, and then with this information to make a choice from the likely outcomes of different possible series of actions. If in contrast choices were made only on the basis of the reinforcement value of immediately available stimuli, without the arbitrary syntactic symbol manipulation made possible by language, then the choice strategy would be much more limited, and we might not want to use the term free will, as all the consequences of those actions would not have been computed. It is suggested that when this type of reflective, conscious, information processing is occurring and leading to action, the system performing this processing and producing the action would have to believe that it could cause the action, for otherwise inconsistencies would arise, and the system might no longer try to initiate action. This belief held by the system may partly underlie the feeling of free will. At other times, when other brain modules are initiating actions (in the implicit systems), the conscious processor (the explicit system) may confabulate and believe that it caused the action, or at least give an account (possibly wrong) of why the action was initiated. The fact that the conscious processor may have the belief even in these circumstances that it initiated the action may arise as a property of it being inconsistent for a system that can take overall control using conscious verbal processing to believe that it was overridden by another system. This may be the reason why confabulation occurs.

The interesting view we are led to is thus that when probabilistic choices influenced by stochastic dynamics are made between the implicit and explicit systems, we may not be aware of which system made the choice. Further, when the stochastic noise has made us choose with the implicit system, we may confabulate and say that we made the choice of our own free will, and provide a guess at why the decision was taken. In this scenario, the stochastic dynamics of the brain plays a role even in how we understand free will (Rolls and Deco, 2010; Rolls, 2011, 2012).

## The precuneus, the sense of self and agency, and its disturbance in schizophrenia

Further light on the different brain systems involved in different aspects of decision-making and willed action (Rolls, 2013) is provided by schizophrenic patients in which the sense of agency and willed action is disturbed (Pu et al., 2013). We have found using connectivity analyzes on resting state fMRI measurements that the largest alteration in schizophrenic patients vs. controls in the functional connectivity (measured by the correlation between the activations in different brain regions) was a weakened coupling between the posterior cingulate gyrus and precuneus (Pu et al., 2013). The magnitude of the decrease in this coupling was found to be positively correlated with the disturbance in volition, that is in the willful initiation, sustenance, and control of thoughts, behavior, movements, and speech, in subsequent correlation analyzes [This is G13 in the PANSS (positive and negative syndrome scale for schizophrenia) Kay et al., 1987]. Further, morphometric analysis identified reduced gray matter volume in the precuneus. The disturbance in the sense of will, self, and agency, prominent symptoms of schizophrenia, may thus be related to the reduced functioning of the precuneus and posterior cingulate cortex (Pu et al., 2013).

These findings are of interest, for the precuneus has been described (Cavanna and Trimble, 2006) as being involved in “visuo-spatial imagery, episodic memory retrieval (which essentially always has a spatial component Rolls, 2010), and in self-processing operations namely first-person perspective taking and an experience of agency.” Further, the precuneus is described as being involved in self-consciousness, and as having high activity when humans are engaged in self-related mental representations during rest. The activity in the precuneus and posterior cingulate areas decreases during engagement in non-self-referential goal-directed actions, that is when actions are performed under the control of external stimuli, and are not self-generated (Cavanna and Trimble, 2006). The precuneus and posterior cingulate cortex are thus part of the default-mode network (Raichle et al., 2001), which is active when subjects are not initiating actions to external stimuli.

These findings, and previous research on the precuneus and its connected areas (Cavanna and Trimble, 2006), thus lead me to propose that the reduced sense of agency and that the self is in control, which is a key symptom of schizophrenia (Sass and Parnas, 2003), is related to the reduction in the coupling between the precuneus and the posterior cingulate cortex, identified in both patient groups in the investigation (Pu et al., 2013). Consistent with this proposal, the intrinsic activity of the brain during the resting-state is thought to reflect self-referential processing, which is reduced in schizophrenia, and resting state measurements in schizophrenic patients with fMRI and positron emission tomography showed hypoactivation compared to controls in the precuneus, posterior cingulate cortex, and left hippocampus (Kuhn and Gallinat, 2011). Further, the concept that misattributions of agency in schizophrenia are due to impaired predictions concerning the sensory consequences of one's own actions (Synofzik et al., 2010) also fits the view that the precuneus and posterior cingulate cortex process self-generated visuo-spatial information (Cavanna and Trimble, 2006).

Thus, the precuneus and other parts of the default mode network appear to be involved in self-initiated action in contrast to externally triggered action, and weakened functional connectivity in this system appears to be related to disturbances in volition, the sense of self, and agency that are found in for example schizophrenia (Pu et al., 2013).

## Conclusions

The evidence reviewed here suggests that there are multiple decision-making systems in the brain, and multiple routes to action, only some of which involve self-initiated in contrast to externally triggered action, and only some of which involve conscious processing (Rolls, 2005b, 2008a, 2011, 2013). The evidence has important implications for understanding the initiation of willed actions, and our reports about why actions are performed, and about when they are initiated by decision-making processes.

### Conflict of interest statement

The author declares that the research was conducted in the absence of any commercial or financial relationships that could be construed as a potential conflict of interest.
